# Cement Leakage in Cement-Augmented Fenestrated Pedicle Screws for Osteoporotic Spine: Risk Stratification with Quantitative Computed Tomography Analysis

**DOI:** 10.3390/jcm14228178

**Published:** 2025-11-18

**Authors:** Akira Shinohara, Tomoaki Kanai, Shunsuke Katsumi, Shintaro Obata, Hiroki Wakiya, Takero Tsuzuki, Mitsuru Saito

**Affiliations:** Department of Orthopedic Surgery, Jikei University School of Medicine, 19-18 Nishi-Shimbashi, Minato-Ku, Tokyo 105-8471, Japan; tkanai@jikei.ac.jp (T.K.); shnskktsm@jikei.ac.jp (S.K.); r1md-wakiya@jikei.ac.jp (H.W.); h25ms-tsuzuki@jikei.ac.jp (T.T.); xlink67@jikei.ac.jp (M.S.)

**Keywords:** pedicle screws, bone cement, osteoporosis, spinal fusion, computed tomography, risk factors

## Abstract

**Background/Objectives**: Cement-augmented fenestrated pedicle screws (CAFPSs) are widely used to enhance fixation strength in osteoporotic vertebrae; however, cement leakage remains a major concern because it can lead to severe complications. This study aimed to clarify the frequency, patterns, and risk factors of cement leakage using postoperative computed tomography (CT). **Methods**: A total of 302 screws placed in 79 osteoporotic patients who underwent spinal fixation with CAFPSs between March 2022 and December 2024 were retrospectively analyzed. Cement leakage was evaluated using postoperative CT, and risk factors were examined by logistic regression and receiver operating characteristic (ROC) curve analysis. **Results**: Cement leakage was observed in 46 patients (58.2%) and 71 screws (23.5%), but no severe complications such as symptomatic pulmonary embolism occurred. Multivariate analysis identified right-sided screw insertion (OR = 2.498, 95% CI: 1.270–4.913, *p* = 0.008) and shorter lateral cortical wall distance (OR = 0.547, 95% CI: 0.469–0.638, *p* < 0.001) as independent risk factors. ROC curve analysis demonstrated high predictive accuracy for lateral cortical wall distance (area under the curve = 0.842), with a cutoff value of 9.21 mm (sensitivity = 0.845; specificity = 0.719). Cement leakage occurred significantly more frequently in the thoracic spine than in the lumbar spine (34.2% vs. 17.0%, *p* < 0.001). **Conclusions**: Right-sided screw insertion and shorter lateral cortical wall distance were identified as major risk factors for cement leakage with CAFPSs. Quantitative CT-based assessment may contribute to risk stratification and optimization of screw placement planning to improve surgical safety.

## 1. Introduction

As population aging progresses, the number of patients with osteoporosis has been steadily increasing, accompanied by a marked rise in fragility fractures and demand for spinal fusion surgery [1]. Posterior fixation using pedicle screws is widely recognized as a standard technique for achieving spinal stability [2]. However, in osteoporotic vertebrae, reduced bone mineral density decreases screw fixation strength, often leading to postoperative loosening, screw pull-out, and an increased risk of revision surgery [3,4,5].

To address this issue, cement-augmented fenestrated pedicle screws (CAFPSs), which allow the injection of polymethylmethacrylate (PMMA) through fenestrations in the screw shaft to enhance fixation within the vertebral body, have been developed. CAFPSs have shown high efficacy, particularly in elderly patients with osteoporosis, and numerous studies have reported significant reductions in screw loosening and pull-out compared with conventional screws [6,7,8].

In Japan, the Japanese Society for Spine Surgery and Related Research has taken the lead in defining the indications and usage guidelines for CAFPSs, promoting careful introduction of the technique. As a result, the clinical application of CAFPSs in Japan has evolved in a unique manner compared with other countries. This study sought to clarify the early outcomes of CAFPS introduction at our institution.

Despite the careful introduction of CAFPSs, cement leakage associated with CAFPSs is a unique complication. Because cement can flow along unpredictable paths within fragile cancellous bone, it may extravasate beyond the vertebral body [9,10]. In particular, leakage into the major venous system can cause pulmonary embolism or cardiovascular complications, representing a critical clinical problem [9]. Cement leakage has been classified into three types: (1) leakage into segmental veins, (2) extravasation into the epidural space, and (3) extrusion as a cement mass outside the vertebral body [11]. However, the reported incidence and clinical significance vary, and no standardized evaluation criteria have been established.

Potential risk factors for cement leakage include vertebral bone mineral density and Hounsfield unit (HU) values as surrogates [12], screw length, diameter, insertion angle, injection volume and viscosity, and the presence of cortical defects [11,13]. However, these factors are difficult to evaluate and control objectively and in real time during surgery, highlighting the need for practical quantitative indicators applicable in clinical practice.

In this study, postoperative computed tomography (CT) images from patients with osteoporosis who underwent spinal fixation with CAFPSs were retrospectively analyzed to evaluate the incidence of and risk factors for cement leakage, with particular focus on anatomical parameters that could be objectively and quantitatively assessed intraoperatively, including vertebral level, screw insertion side (right vs. left), and lateral cortical wall distance. Although several studies have investigated the risk factors for cement leakage in cement-augmented pedicle screw fixation, most analyses have relied on qualitative assessment or categorical variables. In contrast, this study used computed tomography (CT)–based quantitative measurements, such as the lateral cortical wall distance, to predict cement leakage risk. This approach provides an objective, reproducible parameter for preoperative risk evaluation, which has not been clearly quantified in previous studies. The purpose of this study was to clarify the frequency and patterns of cement leakage associated with CAFPSs, to identify quantitative risk factors that may assist intraoperative decision-making, and to provide fundamental data that could contribute to navigation support, integration with artificial intelligence (AI) technologies, and the establishment of safer surgical techniques.

## 2. Materials and Methods

### 2.1. Study Design and Ethical Approval

This retrospective, observational study involved consecutive cases treated between March 2022 and December 2024 at Jikei University Hospital and one affiliated institution. All procedures were performed by multiple surgeons belonging to the same spine surgery group, all of whom were board-certified orthopedic surgeons with expertise in spinal surgery. The surgeries were conducted under standardized techniques and protocols, thereby minimizing variability. This study was approved by the Institutional Review Board of Jikei University Hospital (initial approval date: 2 July 2018; extended approval date: 10 June 2025).

### 2.2. Patient Selection and Inclusion Criteria

This study included 79 patients with osteoporotic vertebral fractures or degenerative spinal diseases associated with osteoporosis-related bone fragility, who underwent posterior spinal fixation using CAFPSs. A total of 302 screws were analyzed. Postoperative CT was performed in all cases for evaluation.

The following variables were assessed: age, sex, screw length (mm), screw diameter (categorical variable), screw laterality (right/left), screw placement accuracy, lateral cortical wall distance (mm), HU values as a surrogate for bone mineral density, vertebral level of screw insertion, cement injection volume, and the presence or absence of cement leakage. HU values were measured on preoperative CT images according to previous reports [14]. A region of interest (ROI) was placed in the cancellous bone at the mid-vertebral body of the instrumented level, avoiding the cortical bone, and the mean HU value was recorded. Screw diameter and cement volume were obtained from operative records. Cement was injected with a target volume of 1 mL per screw under careful monitoring with lateral fluoroscopy. If cement leakage was suspected, injection was immediately discontinued. The presence of cement leakage was finally assessed by postoperative CT in all cases.

### 2.3. Surgical Procedure and Cement Injection Protocol

The screw systems used were the Expedium Verse or Viper Prime Fenestrated Screw System with Vertecem V+ cement (DePuy Synthes, Raynham, MA, USA). CAFPSs were applied only in cases with osteoporosis requiring posterior fixation of ≥2 levels, with a maximum of 2 vertebrae (4 screws) per patient, in accordance with the “Guidelines for Fenestrated Cement-Augmented Pedicle Screw Use” established by the Japanese Society for Spine Surgery and Related Research during the study period. Screw placement was limited to thoracic and lumbar vertebrae.

Polymethylmethacrylate (PMMA) was used as the bone cement. After mixing, an approximately 10 min waiting period was allowed for the cement to reach an appropriate viscosity. Viscosity was assessed subjectively at each procedure by tapping the cement with gloves. If insufficient viscosity was suspected (e.g., excessive fluidity), injection was further delayed until optimal consistency was reached to minimize the risk of leakage.

### 2.4. CT Evaluation and Definition of Cement Leakage

Cement leakage was evaluated on postoperative CT images obtained within 3 days after surgery. Leakage was classified according to a modified Yeom classification [7,11].

### 2.5. Quantitative Measurements

**Figure 1:**
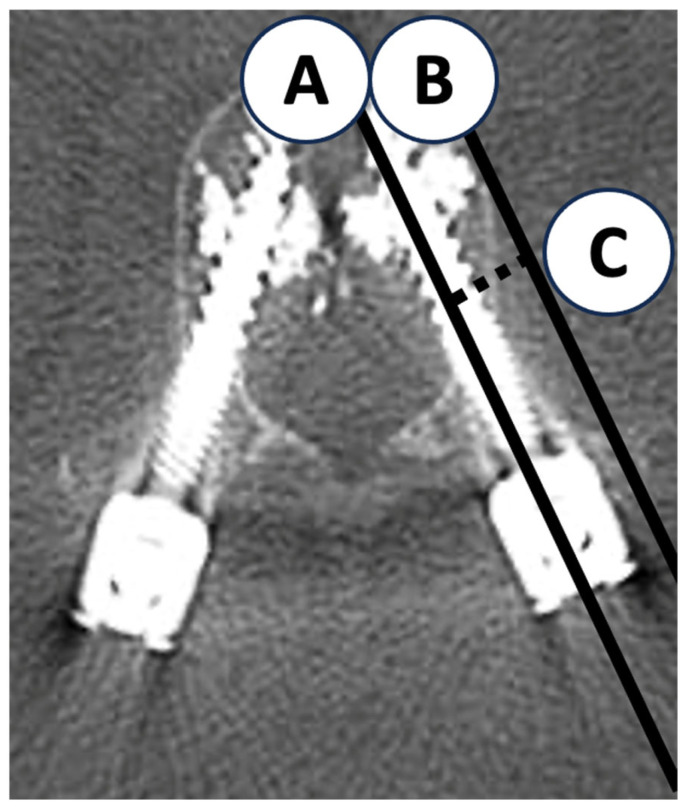
Measurement method of lateral cortical wall distance. A: Screw axis (solid line); B: Tangent line to the lateral cortical wall parallel to the screw (solid line); C: Lateral cortical wall distance (distance between A and B (dotted line).

For quantitative assessment of the spatial relationship between pedicle screws and the vertebral lateral cortex, the lateral cortical wall distance was measured. Because metal artifacts and the high density of cement often reduced screw visibility in CAFPSs, the distance was defined as the shortest perpendicular line from the screw axis to the lateral cortical wall on axial CT images (Figure 1). To minimize measurement error due to variability in cement morphology and image clarity, all measurements were performed by two orthopedic surgeons and finalized by consensus. This method ensured reproducibility and clinical validity of the measurements.

### 2.6. Statistical Analysis and Sample Size Estimation

Statistical analysis was performed using IBM SPSS Statistics version 22.0 (IBM Japan, Ltd., Tokyo, Japan). Because this was a retrospective study, no a priori sample size calculation was performed, and all eligible cases during the study period were included. The final sample of 302 screws (71 events) was sufficient to detect an assumed odds ratio of 2.0 at α = 0.05 with 80% power, and the events-per-variable (EPV) value of 14.2 exceeded the recommended threshold of 10. Analyses were conducted at three levels: screw, vertebral body, and patient. Univariate logistic regression analysis was performed first. Vertebral level was analyzed both as an ordinal variable (Th7–L5 per one level) and as a binary variable (thoracic vs. lumbar). Variables with *p* < 0.2 were included in the multivariate logistic regression analysis (forced entry method), and adjusted odds ratios (ORs) with 95% confidence intervals (CIs) were calculated. A *p* value < 0.05 was considered significant. Receiver operating characteristic (ROC) curve analysis was applied to continuous variables (lateral cortical wall distance and HU values) to calculate sensitivity, specificity, and area under the curve (AUC). The optimal cutoff value for lateral cortical wall distance was determined using the Youden index. Interaction between screw laterality (right vs. left) and lateral cortical wall distance was assessed by constructing logistic regression models with interaction terms, with *p* > 0.05 considered not significant. Collinearity was evaluated using the variance inflation factor (VIF) and tolerance; a VIF < 2 was interpreted as negligible collinearity.

## 3. Results

### 3.1. Patient and Screw Characteristics

In this study, 302 screws were inserted in 79 patients (65 female, 14 male) who underwent spinal fixation with CAFPSs. Patient demographics are summarized in Table 1. The mean age was 76.8 ± 8.38 years. The mean screw diameter was 6.45 ± 0.71 mm, and the mean screw length was 43.69 ± 3.49 mm. No screw malposition was observed, and no severe complications, including symptomatic pulmonary embolism, were encountered. Postoperative CT was used to analyze cement leakage and related factors. Screw distribution by vertebral level was as follows: Th7 2, Th8 4, Th9 6, Th10 28, Th11 38, Th12 36, L1 28, L2 52, L3 52, L4 36, and L5 20. The most frequent sites of screw placement were L2 and L3 (52 screws each), with the majority of CAFPSs used from the thoracolumbar junction to the lower lumbar spine. The mean cement injection volume per screw was 0.96 ± 0.17 mL.

### 3.2. Frequency and Distribution of Cement Leakage

**Figure 2:**
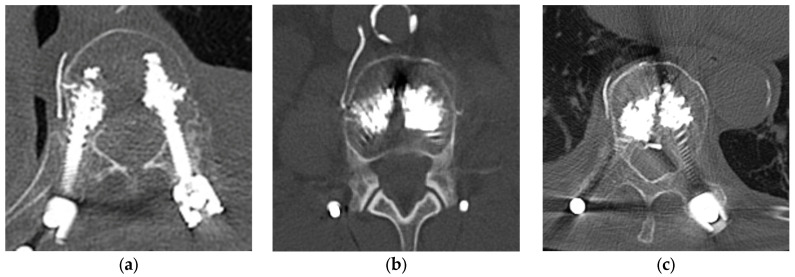
Representative CT images of the cement leakage classification. (**a**) Leakage into the segmental vein (Type S); (**b**) Leakage into the inferior vena cava (Type S); (**c**) Leakage into the segmental vein and the spinal canal (Type S + B).

Cement leakage was observed in 46 of 79 patients (58.2%) and in 71 of 302 screws (23.5%). All cases involved leakage into segmental veins (Type S). Of them, leakage into the major venous system was noted in 2 screws (0.7%): one into the inferior vena cava (IVC) and one into the azygos vein, both via right segmental veins. In addition, combined leakage into the spinal canal (Type B) was observed in 3 screws (0.99%), which were therefore classified as Type S + B (Figure 2). Distribution of Type S leakage was as follows: Th8 2 (50%), Th9 4 (66.7%), Th10 8 (28.6%), Th11 14 (36.8%), Th12 11 (30.6%), L1 5 (17.9%), L2 10 (19.2%), L3 9 (17.3%), L4 6 (16.7%), and L5 2 (10.0%). Type S + B was identified in one screw at Th10 and one at L2. No cases of isolated Type B leakage were observed. For Type S, any cement distributed along vascular structures or extending beyond the vertebral cortex in continuity with segmental veins or the paravertebral venous plexus was defined as venous leakage, regardless of volume or extent.

### 3.3. Univariate and Multivariate Logistic Regression Analyses

Univariate logistic regression identified shorter lateral cortical wall distance as the strongest factor associated with cement leakage (OR = 0.582, 95% CI: 0.506–0.669, *p* < 0.001). Screw laterality (right vs. left) and screw length also met the threshold of *p* < 0.2 and were included in multivariate logistic regression (Table 2). Multivariate analysis identified right-sided screw insertion and lateral cortical wall distance as independent risk factors for cement leakage. Right-sided screws had an approximately 2.5-fold higher risk of leakage compared with left-sided screws (OR = 2.498, 95% CI: 1.270–4.913, *p* = 0.008). Each 1 mm increase in lateral cortical wall distance reduced the risk of leakage by approximately 45% (OR = 0.547, 95% CI: 0.469–0.638, *p* < 0.001).

### 3.4. Receiver Operating Characteristic Curve Analysis at the Screw Level

**Figure 3:**
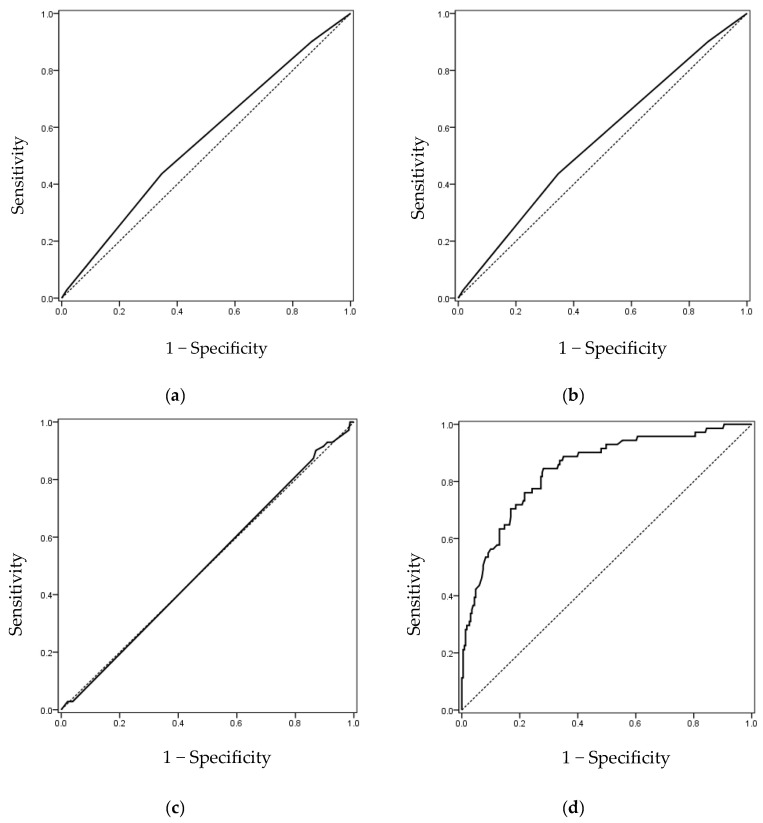
Receiver operating characteristic curve analyses for the presence versus absence of cement leakage at the screw level. (**a**) Screw diameter (AUC = 0.555), (**b**) screw length (AUC = 0.552), (**c**) cement volume (AUC = 0.502), and (**d**) lateral cortical wall distance (AUC = 0.842). Only lateral cortical wall distance demonstrates significant discriminative ability. Detailed statistics are presented in Table 3. Abbreviations: ROC, receiver operating characteristic; AUC, area under the curve.

ROC curve analysis was performed for screw diameter, screw length, cement volume, and lateral cortical wall distance (Table 3). Screw diameter (AUC = 0.555), screw length (AUC = 0.552), and cement volume (AUC = 0.502) all demonstrated limited predictive value (AUC < 0.6). In contrast, lateral cortical wall distance showed high predictive accuracy (AUC = 0.842, 95% CI: 0.789–0.896, *p* < 0.001), with an optimal cutoff value of 9.21 mm (Figure 3).

### 3.5. Analysis at the Vertebral Level (HU Values)

Because dual-energy X-ray absorptiometry (DXA) was not available for all cases, HU values obtained from CT images were used as a surrogate marker of bone mineral density. On both univariate and multivariate analyses, HU values were not significantly associated with cement leakage (OR = 1.072 per 10 HU, 95% CI: 0.992–1.158, *p* = 0.077). ROC curve analysis yielded an AUC of 0.594, although the cutoff value of 51.15 showed high sensitivity (0.933) and low specificity (0.272).

### 3.6. Analysis at the Patient Level (Age and Sex)

At the patient level, univariate analysis suggested weak associations between age (OR = 1.045, 95% CI: 0.993–1.099, *p* = 0.087) and sex (female: OR = 1.759, 95% CI: 0.874–3.541, *p* = 0.113) with cement leakage; however, neither was significant. Multivariate analysis similarly did not identify age or sex as independent risk factors. ROC curve analysis for age yielded an AUC of 0.623 (95% CI: 0.495–0.750, *p* = 0.065), with a cutoff value of 72.5 years (sensitivity = 0.851; specificity = 0.375), indicating only modest predictive accuracy.

### 3.7. Interaction and Collinearity Analysis

Evaluation of the interaction between screw laterality and lateral cortical wall distance showed no significant interaction in the multivariate logistic regression model (*p* = 0.829). Collinearity analysis showed VIF = 1.009 and tolerance = 0.991, both within acceptable limits. Thus, both factors were considered independent risk factors. However, given the limited sample size, interaction effects cannot be entirely excluded, and further validation in larger cohorts is warranted.

### 3.8. Analysis by Vertebral Level

Univariate logistic regression analysis demonstrated that vertebral level was significantly associated with cement leakage, both when analyzed as an ordinal variable (per one level: OR = 0.820, 95% CI: 0.728–0.923, *p* = 0.001) and as a binary variable (lumbar vs. thoracic: OR = 0.394, 95% CI: 0.229–0.679, *p* = 0.001). These results suggested that higher vertebral levels, particularly the thoracic spine, carried a greater risk of leakage.

However, after adjusting for screw laterality and lateral cortical wall distance on multivariate analysis, vertebral level was no longer a significant independent risk factor (per one level: OR = 1.004, *p* = 0.962; lumbar vs. thoracic: OR = 1.059, *p* = 0.877). This indicates that vertebral level itself was not an independent predictor, but was likely confounded by laterality and screw position.

In additional multivariate models including an interaction term between vertebral level and screw laterality, no significant interaction was observed (*p* = 0.740 and *p* = 0.907). Furthermore, leakage rates did not differ significantly between right- and left-sided screws at each vertebral level.

**Figure 4:**
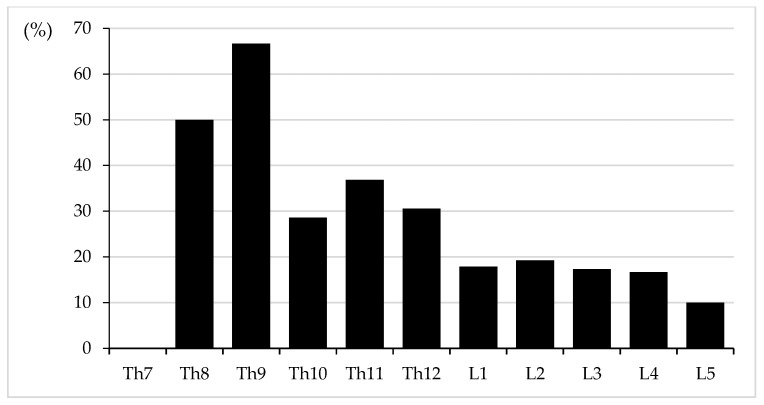
Cement leakage rates (%) by vertebral level from Th7 to L5. The y-axis indicates the percentage of screws with cement leakage at each vertebral level. The Cochran–Armitage trend test shows a significant decreasing trend from the thoracic to the lumbar spine (*p* < 0.001).

Detailed analysis by vertebral level (Table 4) showed that cement leakage occurred in 34.2% of thoracic screws (39/114) and 17.0% of lumbar screws (32/188), with a significantly lower incidence in the lumbar spine. By individual vertebra, leakage rates were highest in upper thoracic levels such as Th9 (66.7%), Th8 (50.0%), and Th11 (36.8%), but lowest at L5 (10.0%). Trend analysis using the Cochran–Armitage test confirmed a significant decreasing trend in the leakage rate with lower vertebral levels (*p* for trend < 0.001) (Figure 4).

Taken together, these results indicate that, although vertebral level influences cement leakage, it is not an independent predictor, and leakage risk is more strongly determined by intraoperative factors and anatomical relationships such as screw laterality and lateral cortical wall distance.

## 4. Discussion

The primary pathway of cement leakage is through the vertebral venous plexus, which lacks venous valves and therefore facilitates retrograde flow or extraosseous leakage when excessive injection or elevated intravertebral pressure occurs [3]. In particular, the lumbar vertebral venous plexus communicates directly with the inferior vena cava (IVC), creating a structural predisposition for intravascular leakage in the presence of excessive injection or elevated pressure [6]. According to Yeom’s classification, Type S leakage into segmental veins is the most frequent subtype [11], and Guo et al. have reported an incidence ranging from 61% to 76% [7,9,13]. Intravascular leakage can cause severe complications such as pulmonary embolism or cardiac perforation, as demonstrated by multiple case reports [15,16,17,18,19,20,21], underscoring the necessity for preventive strategies.

In the present study, the risk factors for cement leakage during cement-augmented fenestrated pedicle screw (CAFPS) fixation in osteoporotic patients were retrospectively analyzed. Multivariate analysis identified right-sided screw insertion and shorter lateral cortical wall distance as independent risk factors. Notably, lateral cortical wall distance showed high predictive accuracy, with an AUC of 0.842 on ROC curve analysis, and the clinically applicable cutoff was determined to be 9.21 mm. The leakage risk increased markedly below this threshold, suggesting that shorter lateral cortical wall distance is a critical anatomical determinant of cement migration. Both factors can be readily assessed preoperatively using CT imaging, providing objective and quantifiable parameters for risk stratification in CAFPS procedures. In contrast to the predominantly subjective assessments used in previous studies, the present findings highlight the clinical value of establishing quantitative, reproducible criteria. These findings suggest that the HU value has limited clinical utility as a predictor of cement leakage compared with lateral cortical wall distance, indicating that anatomical parameters may better reflect true leakage risk than bone density alone.

The present results are consistent with recent large-scale studies. Guo et al., in a cohort of 202 patients and 950 screws, reported that most cases of cement leakage were Type S or Type B, and they identified right-sided screws, greater cement volume, and anterior screw placement as independent risk factors [7]. Similarly, a multicenter Japanese study by Takahashi et al. found a leakage rate of 31.3%, with risk factors including right-sided screws, thoracic level, larger screw diameter, and cement volume ≥ 1.5 mL [22], in line with the present finding of higher leakage risk for right-sided and thoracic screws. Liu et al. demonstrated that thoracic CAFPSs increased the risk of Type S leakage and right-heart complications [9], whereas Janssen et al. reported that greater screw number was associated with pulmonary embolism [23]. In the present series, cement volume was limited to 1 mL per screw, and injection was discontinued immediately upon fluoroscopic evidence of vascular leakage. In contrast to these previous studies, which primarily analyzed categorical or qualitative parameters such as screw number, diameter, and vertebral level, the present study incorporated a CT-based quantitative evaluation using the lateral cortical wall distance. This parameter showed a strong independent association with cement leakage and demonstrated high predictive accuracy (AUC = 0.842). The introduction of this quantitative metric provides an objective and reproducible approach to risk assessment, thereby enhancing the predictive precision and clinical applicability of CAFPS procedures. To the best of our knowledge, no previous study has proposed a validated CT-derived cutoff value for cement leakage risk, highlighting the novelty and clinical significance of the present findings. This strict protocol resulted in a leakage rate of 23.5% at the screw level. Importantly, despite rigorous CT-based evaluation that identified even small leakages, clinically significant leakage into the major venous system (azygos vein or IVC) was observed in only 2 screws (0.7%), and no cases of cardiac perforation or symptomatic Type B leakage were recorded.

The increased risk of leakage with right-sided screws may be explained by the anatomical and hemodynamic asymmetry of the vertebral venous system. In the lumbar region, right segmental veins are shorter and drain directly into the IVC [24]. Since the IVC lacks valves, respiratory fluctuations create negative intrathoracic pressure, predisposing injected cement to retrograde migration from right-sided screws into the venous system [7,24]. In the thoracic region, the azygos vein, located on the right side, communicates with the vertebral venous plexus and drains into the superior vena cava. Similar to the IVC, the azygos system lacks valves, permitting bidirectional flow. Consequently, injection pressure can facilitate retrograde cement migration, particularly given the close communication between the vertebral venous plexus and the azygos system [25,26]. From a fluid dynamics perspective, based on Poiseuille’s law, larger-caliber vessels such as the IVC and azygos vein allow greater flow, thereby increasing the likelihood of cement migration [26,27]. Consistently, cases of cardiac perforation reported by Kim, Takahashi, and others were associated with right-sided venous leakage [19,20,22].

Thoracic vertebrae are also anatomically smaller and have higher venous density than lumbar vertebrae [9,28], which may explain the higher leakage rates observed at thoracic levels. Taken together, these anatomical and physiological features support our findings that right-sided screws and thoracic levels constitute risk factors for cement leakage.

Importantly, the two risk factors identified in the present study, right-sided screw insertion and lateral cortical wall distance < 9.21 mm, are quantifiable using preoperative CT. Lateral cortical wall distance, in particular, could be incorporated into screw trajectory planning, navigation-assisted surgery, and simulation systems to improve safety margins. Furthermore, the cutoff value of 9.21 mm is suitable for integration into AI-assisted navigation platforms, enabling real-time intraoperative risk detection and visualization. This integration of quantitative radiological parameters with emerging technologies may contribute to safer and more rational surgical strategies. In addition, the strict injection protocol, limiting cement to 1 mL per screw and halting injection upon fluoroscopic detection of vascular spread, demonstrated that practical measures can effectively mitigate leakage risk, particularly severe complications such as Type B or intravascular leakage.

The present study has several methodological strengths. First, postoperative CT was performed in all cases, enabling objective and high-resolution assessment of cement leakage at the screw level, surpassing previous studies that relied on plain radiographs or partial CT evaluation. This ensured detection of subtle leakage types, thereby improving the reliability of risk analysis. Second, emphasis was placed on preoperative CT-based parameters such as screw laterality and lateral cortical wall distance, providing clinically applicable quantitative markers with potential for integration into navigation and AI systems. Third, procedures were performed under a uniform protocol with standardized cement volume, viscosity management, and injection cessation criteria, minimizing operator-related bias.

Nevertheless, the present study also has limitations. It was a retrospective analysis conducted under a single standardized protocol, and external validity requires verification in multicenter and prospective settings. Although all procedures were performed under a standardized protocol, the surgeries were conducted by multiple surgeons. Minor variations in surgical technique, cement injection timing, and viscosity judgment could have affected the leakage rate. Therefore, potential operator-dependent variability should be acknowledged as a study limitation. The study population was restricted to osteoporotic patients, and extrapolation to traumatic or neoplastic cases should be approached with caution. Furthermore, intraoperative factors such as cement viscosity, injection pressure, and insertion angle were not sufficiently assessed. Finally, limiting cement volume to 1 mL per screw precluded evaluation of the relationship between leakage risk and fixation strength at higher cement volumes. However, biomechanical studies suggest that higher cement volumes do not necessarily improve pull-out strength: Weiser et al. reported no significant difference between 3 mL and 1 mL injections [29], whereas Frankel et al. demonstrated increased pull-out strength (119–162%) with cement augmentation, but no significant difference between 2.8 mL and 5.5 mL [30]. These findings indicate that excessive cement injection does not confer additional biomechanical benefit and may unnecessarily increase leakage risk. The present standardized protocol of approximately 1 mL per screw therefore represents a balanced, reproducible approach optimizing both safety and fixation strength. Notably, the lack of correlation between cement leakage and cement volume in the present series can be explained by the strict limitation to ≤1 mL per screw; leakage-prone cases tended to leak from the beginning even with small volumes, whereas exceeding 1 mL would likely increase the risk.

Future studies should focus on validating the quantitative risk indicators identified in this study, particularly the cutoff value for lateral cortical wall distance (9.21 mm) and screw laterality, through multicenter, prospective investigations. Integration of CT-based quantitative assessment with AI-assisted navigation systems may further enhance intraoperative risk prediction and improve clinical applicability. In addition, collaboration with biomechanical research is warranted to optimize the balance of cement volume, fixation strength, and leakage risk.

These quantitative parameters may also be applicable to future AI-based navigation or robotic-assisted spine surgery systems for intraoperative risk visualization and trajectory planning.

In summary, the present study demonstrated the feasibility of predicting and mitigating cement leakage during CAFPS fixation using preoperative CT-based quantitative assessment.

## 5. Conclusions

The present study demonstrated that right-sided screw insertion and lateral cortical wall distance of less than 9.21 mm are independent risk factors for cement leakage during CAFPS fixation in osteoporotic patients. Although vertebral level showed an association on univariate analysis, it was not significant in multivariate analysis, suggesting dependence on other factors. These findings may enhance the accuracy of preoperative risk assessment and provide valuable guidance for navigation-assisted and robotic spine surgery. Future multicenter, prospective studies are warranted to validate these risk factors and to establish standardized strategies for preventing cement leakage in clinical practice.

## Figures and Tables

**Table 1:** Patients’ baseline characteristics.

Variable	Overall (*n* = 79 Patients)
Age, y, mean ± SD	76.8 ± 8.38
Sex, male/female, *n* (%)	14 (17.7)/65 (82.3)
Mean HU value of instrumented vertebrae	84.86 ± 43.59
Instrumented vertebral level, *n* (%)	Thoracic 114 (37.8) Lumbar 188 (62.3)
Number of inserted CAFPSs, *n*	302
Screw diameter, mm, mean ± SD	6.45 ± 0.71
Screw length, mm, mean ± SD	43.69 ± 3.49
Screw laterality, *n* (%)	Right 151 (50.0) Left 151 (50.0)
Cement volume per screw, mL	0.96 ± 0.17

**Table 2:** Univariate and multivariate logistic regression analyses for the presence versus absence of cement leakage at the screw level (*n* = 302).

	Univariate Analysis			Multivariate Analysis		
Variable	OR	95% CI	*p*-Value	OR	95% CI	*p*-Value
Right side (vs. left)	1.503	0.879–2.572	0.137	2.498	1.270–4.913	0.008
Screw diameter (per 1 mm)	0.775	0.532–1.127	0.182	0.856	0.533–1.374	0.519
Screw length (per 5 mm)	0.752	0.511–1.108	0.149	1.511	0.918–2.489	0.105
Cement volume (per 0.1 mL)	1.021	0.881–1.183	0.782	n.e.		
Lateral cortical wall distance (per 1 mm)	0.582	0.506–0.669	<0.001	0.547	0.469–0.638	<0.001

Variables with *p* < 0.2 on univariate analysis were entered into the multivariate model using the forced entry method. Χ^2^ test for coefficients in the multivariate model: *p* < 0.001. Abbreviations: OR, odds ratio; CI, confidence interval; n.e., not entered.

**Table 3:** Receiver operating characteristic curve analysis for each variable.

Variable	AUC	95% CI	*p*-Value	Cut-Off Value	Sensitivity	Specificity
Screw diameter	0.555	0.480–0.630	0.158	≤6.5	0.592	0.532
Screw length	0.552	0.475–0.629	0.187	≤42.5	0.437	0.654
Cement volume	0.502	0.426–0.578	0.967	≤0.85	0.901	0.130
Lateral cortical wall distance	0.842	0.789–0.896	<0.001	≤9.21	0.845	0.719

Abbreviations: ROC, receiver operating characteristic; AUC, area under the curve; CI, confidence interval.

**Table 4:** Cement leakage rates by vertebral level.

	Inserting Screws	Cement Leakage Screws
Th7	2	0 * (0.0)
Th8	4	2 * (50.0)
Th9	6	4 * (66.7)
Th10	28	8 * (28.6)
Th11	38	14 * (36.8)
Th12	36	11 * (30.6)
Thoracic (Th1–12)	114	39 * (34.2)
L1	28	5 * (17.9)
L2	52	10 * (19.2)
L3	52	9 * (17.3)
L4	36	6 * (16.7)
L5	20	2 * (10.0)
Lumbar (L1–5)	188	32 * (17.0)

* (Percentage of cement leakage cases by vertebral level).

## Data Availability

The data that support the findings of this study are not publicly available due to privacy and ethical restrictions. However, de-identified data can be made available from the corresponding author upon reasonable request, in accordance with institutional and ethical regulations.
